# Editorial: Research advances in male fertility: New horizons for investigating human testicular function and development of clinical fertility preservation approaches

**DOI:** 10.3389/fendo.2022.1027813

**Published:** 2022-09-30

**Authors:** Swati Sharma, Jan-Bernd Stukenborg, Stefan Schlatt

**Affiliations:** ^1^ Centre of Reproductive Medicine and Andrology, Institute of Reproductive and Regenerative Biology, University of Münster, Münster, Germany; ^2^ NORDFERTIL Research Lab Stockholm, Department of Women’s and Children´s Health, Karolinska Institutet, and Karolinska University Hospital, Stockholm, Sweden

**Keywords:** testis, fertility preservation, infertility, male reproductive health, germ cells, *in vitro* spermatogenesis

The topic for this special issue on Research Advance in Male Fertility was defined and initiated by the three authors in 2021 inviting submissions to address a broad range of original research papers and reviews. Ten submissions involving 64 authors were published after peer review between October 2021 and July 2022. The accepted two reviews and eight original papers present a wide array of themes and topics describing new and exciting strategies from basic science to clinical applications (**Figure 1**). As of mid-August 2022, the submissions attracted already 12.000 views and 4.000 downloads of the open access submissions.

The early response reveals that male fertility preservation is currently a hot topic in the field of reproductive medicine. This is because poor male reproductive health leads to nearly half of failed pregnancy attempts. Declining semen quality over the past 50 years indicates the role of changing lifestyle and exposure to environmental and toxic components as potential contributing factors. Sperm retrieval is feasible in adult infertile men by using surgical procedures like TESE (Testicular sperm extraction) and can be combined with cryopreserving semen samples for future Assisted Reproductive Technologies (ART) treatments. Prepubertal patients undergoing gonadotoxic treatment to cure malignant or non-malignant diseases or patients with specific genetic abnormalities are at high risk of losing their fertility. Those patients have currently no option for fertility protection or means of fertility preservation to father offspring. Using established strategies, fertility and andrology centers began to cryo-bank immature testis tissue from biopsies to offer fertility preservation strategies that are based on preserving spermatogonial stem cells (SSCs) rather than spermatozoa. While cryobanking occurs, strategies for the generation of gametes from banked tissues do not yet exist for clinical applications but have been developed in animal studies.

This Research Topic focuses therefore on research advances in the field of fertility preservation and the development of strategies to investigate the impact of environmental factors on male reproductive health. A special focus is put also on the development of new experimental strategies to generate male gametes *ex vivo*. The rapidly evolving field is reviewed, and two-dimensional and three-dimensional culture conditions, including organoid and organ culture models, are presented for several species including monkeys.

**Figure 1:**
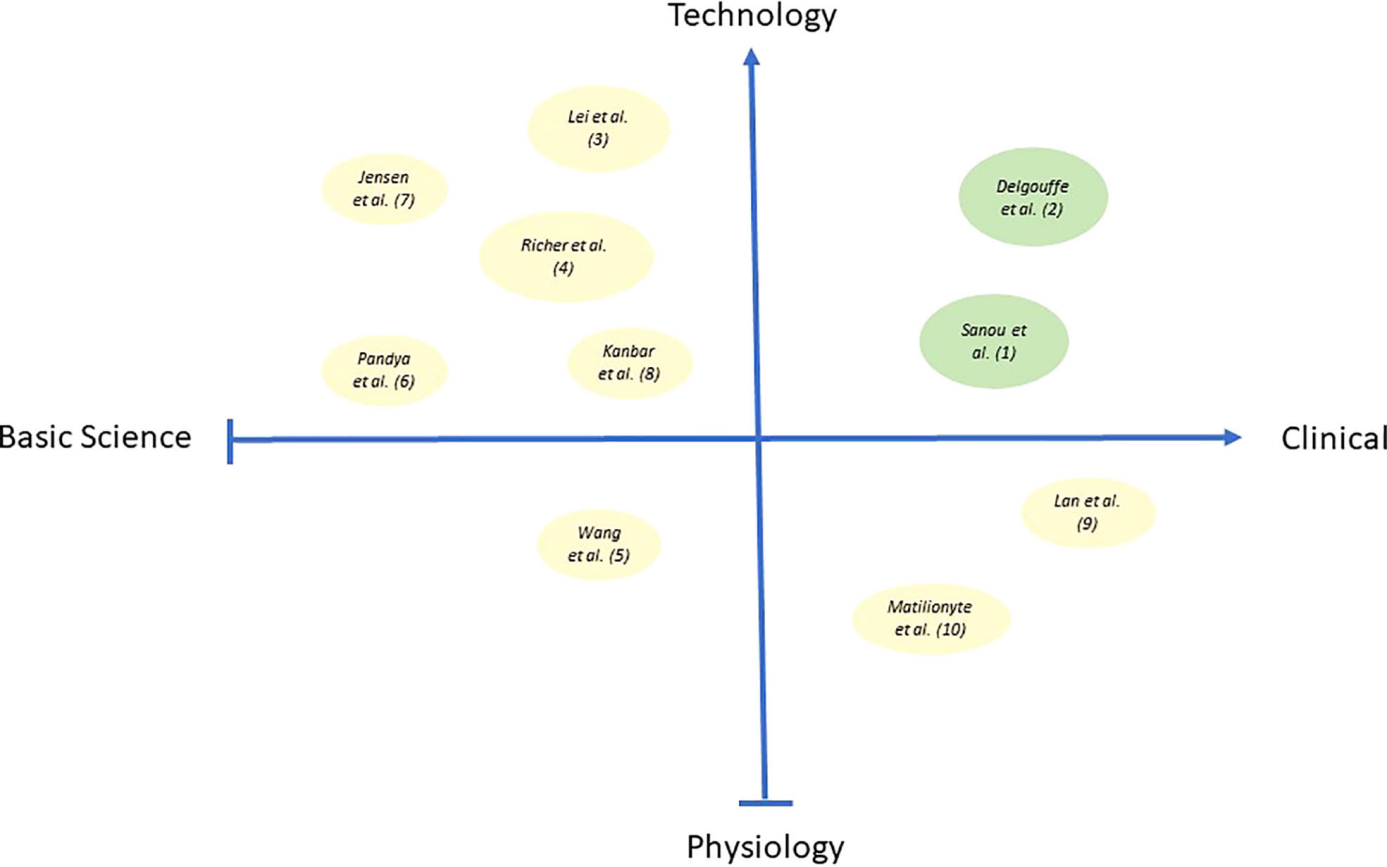
Bubble plot in the figure illustrates the publications from this issue encompassing the quad-spectrum of basic science research, clinical research, physiological and technology-based research. X-axis represents Basic science to Clinical research, and Y-axis represents Physiology to Technology-based research. Bubbles in green color represent the two reviews, and bubbles in pale-yellow color represent eight original manuscripts published as part of this special issue. Different bubble size indicates the impact of specific publications w.r.t citations.

We have observed a mix of submissions covering all aspects from basic science to clinical applications. The submissions cover themes describing novel insights into physiology but also new technical advances and exciting technology. The figure above depicts the range of contents as a plot showing the two reviews (green dots) and eight original research papers (grey dots) in relation to their content. The size of dots relates to the number of citations in each submission indicating the depth of scientific information in each report (**Figure 1**).

Both reviews reveal excellent overviews of the scientific state of the art. Sanou et al. from the Amsterdam University Medical Centre provide an insight into the status of basic research in the area of SSC-based therapies and create an outlook into innovative clinical strategies which can be generated in the future. Although still experimental, biobanking of immature testicular tissue has already become nearly a routine strategy for males facing high gonadotoxic treatments prior to puberty. It is envisaged that the spermatogonia in these cryopreserved samples have the potential of forming spermatids *via* various means and thereby may be used for therapeutic applications later in life. The authors reviewed the past 30 years of research in this field and describe crucial milestones like the transplantation of spermatogonia, grafting of testicular tissue, and different *in vitro* and *ex vivo* culture settings. They claim that it is now time to take the next step in initiating the first clinical applications and trials. Taking into consideration the effectiveness of the SSC-based therapies and addressing hurdles and risks they see safe and effective SSC-based fertility treatments to be implemented in the coming years.

A more clinical perspective on this topic is provided by Delgouffe et al. from the Vrije Universiteit Brussel. Their review addresses the relevant question of inclusion and exclusion criteria for patients when novel treatment options for stem cell-based fertility preservation in boys are established. Today, an increasing number of young patients are at risk of germ cell loss and many centers have started to cryopreserve immature testis tissue. While the methodology was originally considered to be predominantly offered to young cancer patients prior to gonadotoxic chemo- or radiotherapy, it has been realized that banking of testicular tissue is nowadays offered to many patients with non-malignant conditions. These patients arise in the context of conditioning therapy prior to hematopoietic stem cell transplantation (HSCT) and bone marrow transplantation (BMT). A considerable number of patients have genetic or developmental disorders leading to prepubertal germ cell loss and may also be considered for tissue banking. In addition, a significant group of patients is banking after they faced previous exposure to chemo- or radiotherapy. The authors address the question if all patients should be considered for surgical removal as this invasive and still experimental procedure has inherent risks. It could be argued that priority should be given to patients with a significant risk of becoming infertile. The authors review the existing evidence and propose that testicular tissue banking should be offered to young cancer patients in need of high-risk chemo- and/or radiotherapy irrespective of previous low-risk treatment and to patients with non-malignant disorders facing high-risk conditioning therapy. A small group of patients with bilateral cryptorchidism may also be recommended for retrieval and banking. Patients facing medium- to low-risk gonadotoxic exposures usually maintain their fertility and may not be considered for such strategies as are Klinefelter patients for whom the perspectives to undergo routine TESE procedures are more promising than cryobanking of extremely limited SSCs.

Eight original submissions provide significant insights and novel datasets on many significant aspects in the field of male fertility preservation. In the study of Lei et al., the authors considered problems with adequate meiosis in germ cells undergoing spermatogenesis *in vitro*. They express concerns that incomplete synapsis of the homologous chromosomes or impaired homologous recombination may lead to failure of crossover formation and subsequent chromosome nondisjunction. The risk for aneuploid sperm may rise in such *ex vivo* generated gametes. The authors describe the meiotic checkpoints usually eliminating aberrant spermatocytes during spermatogenesis, and present evidence that *in vitro* derived meiotic cells undergo meiosis despite incomplete chromosome synapsis and in the absence of XY-body or crossover formation. They propose to apply an improved *in vitro* system in which the transition of germ cells through various positions in the seminiferous epithelium is mimicked. Despite obvious improvement in meiotic progression, the authors describe that meiotic recombination was still disturbed with only a few meiotic crossovers. Furthermore, they conclude that meiotic checkpoints may not be fully functional under *in vitro* conditions and propose to closely monitor the meiotic process to avoid the generation of aneuploid gametes when such systems are considered for clinical applications.

Shortcomings associated with *in vitro* generation of germ cells are also addressed by Richer et al. Survival of germ cells is limited, and tissue degeneration is prominent in testicular organotypic cultures. The author performed experimental studies generating different 2D and 3D organotypic systems using two different mouse strains. The organoids contained tubule-like structures supporting long-term survival and differentiation of germ cells to the meiotic phase as well as functioning Leydig cell. 3D scaffolds enabled improved spheroidal morphology and generation of functional units revealing both, spermato- and steroidogenesis without the survival of germ cells in long-term culture. Overall, the study implies that further optimization of culture conditions is required. The authors assume that such systems may become valuable tools for studying the interplay of somatic and germ cells and may lead to the development of therapies for male infertility.

Another experimental strategy is described in the study of Wang et al., presenting data on germ cell homing and development following xenotransplantation of human germ cells into the mouse testis. Concerns that *in vitro* propagation of spermatogonia may cause genetic and epigenetic changes prompted the authors to change the strategy and inject germ cell suspensions into the testis without an initial expansion in culture. This strategy may be safer and easier for future clinical applications. Primary cell suspensions from 11 infant testes with cryptorchidism were labeled with green fluorescent dye and directly infused into seminiferous tubules of busulfan-treated mice. Six to nine weeks later whole mount analysis was performed to detect gonocytes and spermatogonia using immunofluorescence staining for MAGEA4, GAGE, UCHL1, SALL4, UTF1, and LIN28 and two somatic cell markers (SOX9 and CYP17A1 for Sertoli and Leydig cells, respectively). Homing of human gonocytes and spermatogonia in mouse seminiferous tubules was observed with a colonization efficiency of approx. 6%. The authors conclude that colonization of mouse seminiferous tubules by human germ cells can be achieved without prior *in vitro* propagation. This observation may simplify the necessary steps required for a successful germ cell transplantation and may reduce the risk of inducing mutations. However, the preliminary observations need to be confirmed and substantiated by additional studies showing that the low number of transplanted SSC is sufficient to secure the presence of sperm in the ejaculate of those patients over time.

Experimental studies exploring how hypothermia during tissue handling prior to organ culture affects the epigenetic integrity of cells in the immature testes were addressed in a study by Pandya et al. The impact of hypothermic holding was analyzed in tissue obtained from 6-day-old mice. As endpoints, relative mRNA expression of DNA methyltransferases Dnmt1, Dnmt3a, and Dnmt3b along with global DNA methylation were analyzed after systematic exposure to 4°C cold medium for 6 and 24 hours. Endpoints were analyzed in mouse testis tissue after two weeks of organotypic culture. No significant variation in methyltransferase expression was detected in relation to the two time periods of exposure. Global DNA methylation was unchanged between 0, 6, and 24 h exposed tissues. Authors conclude that hypothermic holding of immature testis tissue for up to 24 hours does not affect DNA methylation providing experimental evidence that this procedure may also be safe in clinical settings.

Jensen et al. studied the diverse phenotype of testes from infertile men with non-obstructive azoospermia (NOA). These patients show impaired spermatogenesis in dilated and undilated atrophic seminiferous tubules. Active spermatogenesis occurs more frequently in dilated tubules. In this study, attention was focused on the un-dilated tubules and their SSC microenvironment. Analysis of 34 testis tissue samples obtained from undilated areas was performed. Initial analysis revealed hypo-spermatogenesis in five, maturation arrest in 14, and Sertoli cell only in 15 samples. Five control samples from fertile men were also analyzed. Methods to determine the endpoints were routine histology, RT-PCR, and immunofluorescence of germ and Sertoli cell markers. Irrespective of the severity of spermatogenic depletion un-dilated tubules showed an increase in Anti-Müllerian hormone mRNA and protein expression compared to the control. Other markers like GDNF and BMP4 showed variable expression depending on the histological findings at the mRNA and protein level. NOA testes revealed a reduction of germ cell markers DDX4 and MAGE-A4. Changes were also observed in the number of androgen receptor positive Sertoli cells and in mRNA expression. It was concluded that somatic and germ cells are differentially affected indicating different and individualized mechanisms leading to testicular dysfunction. This may require individualized regimens for different damage types when *in vitro* conditions for germ cell production will be defined in the future.

Experimental studies using exciting new microfluidic technology and piglets as model species were performed by Kanbar et al. During the last decades, the successful *in vitro* maturation of immature testicular tissue using organotypic tissue culture in mice has been reported. However, this approach remains challenging in larger mammals and primates. Here the research implemented advances in biomaterial technology creating complex culture systems to better mimic *in vivo* conditions. A comparison of four different organotypic tissue culture systems was performed with a maximal culture period of 30 days. Histological observations and detection of markers provided a large array of endpoints in the tissues. The piglet testis fragments developed all tested systems indicated by the growth of seminiferous tubules, expansion of Sertoli and germ cells, and release of testosterone. While some systems were more efficient, several physiological parameters showed identical progress in all used systems. The authors conclude that microfluidic systems need to be further optimized together with culture media to establish the most favorable conditions for *ex vivo* spermatogenesis in dissected immature testes.

As part of this special topical issue focusing on ‘research advances in fertility preservation’, two original papers were submitted reporting clinical observations. Lan et al. described the success rates when Intra-Cytoplasmic Sperm Injection (ICSI) - Invitro Fertilization (IVF) was performed on patients with non-obstructive azoospermia (NOA). Sperm retrieval rates and ICSI outcomes after micro-TESE in NOA patients remain inconclusive providing no solid base for conducting comprehensive recommendations. The paper reports retrospective data on 968 NOA patients undergoing micro-TESE. Embryological, clinical, and live birth outcomes were analyzed taking various clinical parameters into consideration. Overall sperm retrieval rate was 44.6% enabling to perform ICSI in 299 couples. As an outcome, 150 clinical pregnancies and 140 live-birth deliveries were achieved. Frozen and fresh sperm cycles showed similar outcomes. NOA patients with Azoospermia Factor c (AZFc) microdeletions showed the lowest rate of success. When fewer than 20 sperm were retrieved, the outcome was significantly reduced compared to cycles with more than 20 sperm available for ICSI.

A different clinical dataset was reported by Matilionyte et al., exploring the effects of chemotherapy exposure during pregnancy. The study aimed to describe platinum-based alkylating-like chemotherapeutic exposures on germ cell numbers in the human fetal testis. Specific focus was put on the effects at variable gestational ages of the fetus. The ability to explore such parameters in human fetal testis cultures as a model for chemotherapy exposure during pregnancy may open novel experimental strategies. A total of 23 human fetal testicular tissue samples were available for *in vitro* culture. Three different gestational age groups were defined. Cisplatin or vehicle exposure occurred in culture for 24 hours with analyzing the tissues 72 and 240 hours later. Endpoints analyzed were numbers of gonocytes and (pre)spermatogonia. Depending on gestational age, gonocyte numbers were reduced after 72 hours. At 240 hours post-exposure, gonocytes and spermatogonia showed a reduction in testicular tissues obtained from fetuses in the mid- and late-second trimester, whilst remaining unaffected in early-second trimester tissues. The authors conclude that *in vitro* culture of human fetal testicular tissues presents a promising model system to study the effects of chemotherapy exposure in fetal testes.

In summary, the collection of articles included in this Research Topic on “*Research Advances in Male Fertility: New Horizons for Investigating Human Testicular Function and Development of Clinical Fertility Preservation Approaches*” provides novel and important insights for all researchers and healthcare professionals interested in the field of male reproduction. It will hopefully inspire new investigations and exciting research questions in this important field of testicular research.

## Author contributions

All authors contributed to the drafting, revision, proof-reading and final approval of the editorial.

## Conflict of interest

The authors declare that the research was conducted in the absence of any commercial or financial relationships that could be construed as a potential conflict of interest.

## Publisher’s note

All claims expressed in this article are solely those of the authors and do not necessarily represent those of their affiliated organizations, or those of the publisher, the editors and the reviewers. Any product that may be evaluated in this article, or claim that may be made by its manufacturer, is not guaranteed or endorsed by the publisher.

